# Chromatin interaction analysis reveals changes in small chromosome and telomere clustering between epithelial and breast cancer cells

**DOI:** 10.1186/s13059-015-0768-0

**Published:** 2015-09-28

**Authors:** A. Rasim Barutcu, Bryan R. Lajoie, Rachel P. McCord, Coralee E. Tye, Deli Hong, Terri L. Messier, Gillian Browne, Andre J. van Wijnen, Jane B. Lian, Janet L. Stein, Job Dekker, Anthony N. Imbalzano, Gary S. Stein

**Affiliations:** Department of Cell and Developmental Biology, University of Massachusetts Medical School, 55 Lake Avenue North, Worcester, MA 01655 USA; Program in Systems Biology, University of Massachusetts Medical School, 55 Lake Avenue North, Worcester, MA 01655 USA; Department of Biochemistry and Molecular Pharmacology, University of Massachusetts Medical School, 55 Lake Avenue North, Worcester, MA 01655 USA; Department of Biochemistry & Molecular Biology, Mayo Clinic, 200 First Street SW, Rochester, MN 55905 USA; Department of Biochemistry and University of Vermont Cancer Center, University of Vermont College of Medicine, 89 Beaumont Avenue, Burlington, VT 05405 USA

**Keywords:** Hi-C, Chromosome Conformation Capture, Breast Cancer, Topologically Associating Domain, TAD, Telomere

## Abstract

**Background:**

Higher-order chromatin structure is often perturbed in cancer and other pathological states. Although several genetic and epigenetic differences have been charted between normal and breast cancer tissues, changes in higher-order chromatin organization during tumorigenesis have not been fully explored. To probe the differences in higher-order chromatin structure between mammary epithelial and breast cancer cells, we performed Hi-C analysis on MCF-10A mammary epithelial and MCF-7 breast cancer cell lines.

**Results:**

Our studies reveal that the small, gene-rich chromosomes chr16 through chr22 in the MCF-7 breast cancer genome display decreased interaction frequency with each other compared to the inter-chromosomal interaction frequency in the MCF-10A epithelial cells. Interestingly, this finding is associated with a higher occurrence of open compartments on chr16–22 in MCF-7 cells. Pathway analysis of the MCF-7 up-regulated genes located in altered compartment regions on chr16–22 reveals pathways related to repression of WNT signaling. There are also differences in intra-chromosomal interactions between the cell lines; telomeric and sub-telomeric regions in the MCF-10A cells display more frequent interactions than are observed in the MCF-7 cells.

**Conclusions:**

We show evidence of an intricate relationship between chromosomal organization and gene expression between epithelial and breast cancer cells. Importantly, this work provides a genome-wide view of higher-order chromatin dynamics and a resource for studying higher-order chromatin interactions in two cell lines commonly used to study the progression of breast cancer.

**Electronic supplementary material:**

The online version of this article (doi:10.1186/s13059-015-0768-0) contains supplementary material, which is available to authorized users.

## Background

Three-dimensional genome organization is important for regulation of gene expression by bringing together distant promoter, enhancer and other *cis*-regulatory regions [1–3]. The development of cancer involves several genetic and epigenetic alterations that result in aberrant gene expression [4–7]. Moreover, cancer is a disease characterized by major morphological changes in the nucleus that are used as diagnostic markers [8, 9]. Even though the morphological features of cancer are well characterized, the molecular consequences of the aberrant nuclear morphology are still poorly understood.

The higher-order folding of chromatin within the nucleus involves hierarchical structures spanning different length scales [10]. Microscopic imaging shows that chromosomes are positioned within confined volumes known as chromosome territories [11]. In the nucleus, each chromosome has a preferred, but not fixed, position in which gene-dense chromosomes tend to be at the nuclear interior whereas the gene-poor chromosomes are found near the nuclear periphery [11–14]. Increasing evidence highlights the importance of chromosome and gene positioning during breast cancer initiation [15–17]. Moreover, recent evidence demonstrates the influence of physical spatial proximity in the nucleus on recurrent translocations [18–20].

Several studies have revealed that chromosome territories consist of megabase-scale genomic compartments that are either euchromatic, gene-rich, and highly transcribed (A-type compartments) or heterochromatic, gene-poor, and silent (B-type compartments) [20–23]. The open and closed compartments mostly interact with other open and closed compartments, respectively, whereas there are very few interactions between the two different types of compartments. The open (A-type) compartments preferentially and spatially cluster together in the nuclear interior, whereas the closed (B-type) compartments cluster together near the nuclear periphery [14].

Compartments are composed of 100 kb to 1 Mb scale topologically associating domains (TADs). TADs have been defined as clusters of interactions, in which the enhancers and promoters of co-regulated genes cross-talk with one another. Intra-TAD interactions are much more prevalent than inter-TAD interactions [24]. TADs have been shown to be largely invariant across different species, cell types, and physiological conditions [24, 25] and may act as functional units for transcription regulation [26–28]. Recent work elucidated the role of TADs and transcription factor-associated interactions at a genome-wide level in the context of hormonal regulation (i.e., estrogen or progesterone treatment) [28–35]. TADs are thought to facilitate transcriptional regulation by integrating the regulatory activities within the same domain [10, 26]. Within TADs, looping interactions at the 10 kb to 1 Mb scale bring together enhancers and promoters to regulate gene expression. Functional characterization of long-range interactions in breast cancer has been studied within certain candidate regions [36–40] or by examining the genome-wide interactions of a single locus using more unbiased approaches [41–43]. Probing chromatin structure in cancer has potential as a discovery tool for identifying candidate biomarkers [44], as the organization of the chromatin is often perturbed at different hierarchical levels in cancer [45]. Despite the number of previous studies, differences in genome-wide chromatin structure between normal epithelial cells and tumorigenic breast cancer cells remain unknown.

In this study, in order to characterize different scales of genome organization during breast cancer development, we performed genome-wide chromosome conformation capture (Hi-C) analyses in MCF-10A mammary epithelial and MCF-7 tumorigenic breast cancer cells. Hi-C is a powerful molecular tool to probe genome-wide chromatin interactions in an unbiased way [46]. Our results uncovered fundamental differences of chromatin organization at different genomic scales between two commonly used mammary epithelial and tumorigenic breast cancer cell lines. This work provides an important foundation for understanding the relationship between the alterations in chromatin organization and gene expression in breast cancer.

## Results

### Small, gene-rich chromosomes interact less frequently in the MCF-7 breast cancer genome

In order to probe the genome-wide chromatin structure of mammary epithelial and breast cancer cells, we generated Hi-C libraries from two independent biological replicates for the MCF-10A and MCF-7 cell lines. After sequence filtering [47], a total of ~152 and ~143 million interactions were obtained from the MCF-10A and MCF-7 combined replicate Hi-C libraries, respectively (Figure S1 in Additional file 1), with high reproducibility between the biological replicates (Figure S2 in Additional file 1). For the initial Hi-C analyses, we used the iterative correction method (ICE) [48] to correct for systematic biases, including copy number differences.

**Fig. 1:**
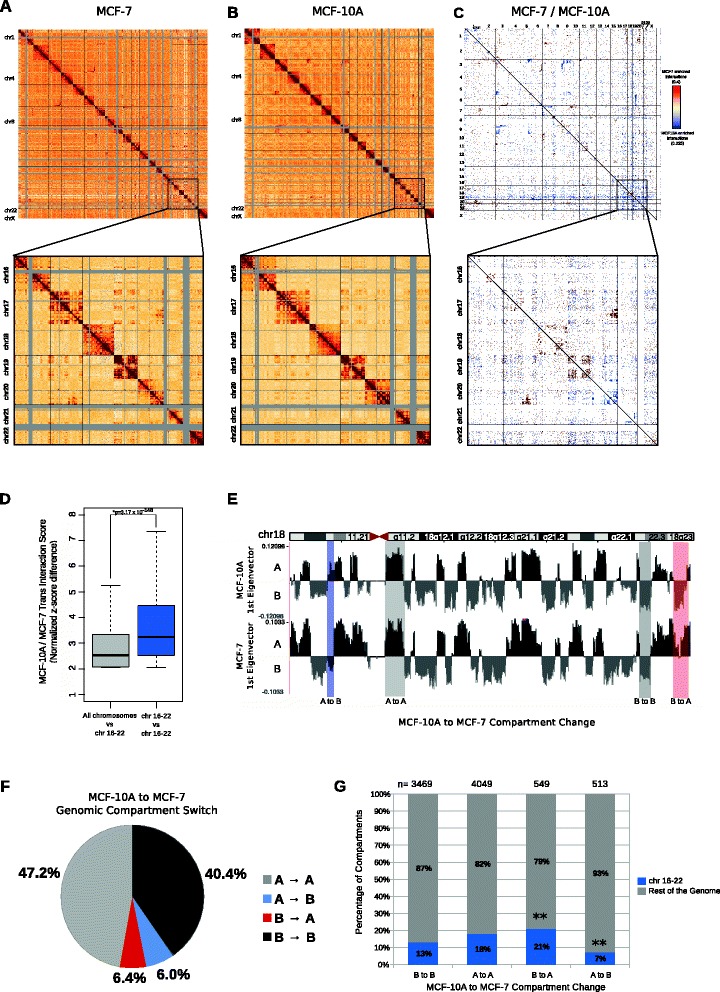
Hi-C analyses identify that small chromosomes (chr16–22) in the MCF10A genome show preferential associations with each other. Genome-wide all-by-all 1-Mb Hi-C interaction heatmap of MCF-10A (**a**) and MCF-7 (**b**) cells. The chromosomes in all-by-all heatmaps are stacked from *top left* to *bottom right* in order (chr1, chr2…chr22 and chrX). The *gray regions* indicate repetitive regions (such as centromeres) in which the sequencing reads could not be mapped. *Intra*-chromosomal interactions were much more frequent than inter-chromosomal interactions. The blocks of enriched inter-chromosomal interactions represent the translocated regions. In the *lower panels*, enlargements of the *cis-* and *trans-*interactions for chr16 through chr22 are shown. **c** Genome-wide heatmap of significant differential interactions between MCF-10A and MCF-7. Each *dot* denotes a genomic region of 6.5 Mb. Chromosomes are stacked from *top left* to *bottom right* from chr1 through chr22 and chrX. The *red color* indicates MCF-7-enriched interactions and the *blue color* indicates MCF-10A-enriched interactions. The *white regions* denote interacting regions that are not significantly changed between the cell lines. In the *lower panel*, significant interactions within and between chr16–22 are shown. **d** Boxplot showing the MCF-10A/MCF-7 inter*-*chromosomal interaction frequency differences between chr16 through chr22 and all the other chromosomes (*grey*) or between chr16 through chr22 (*blue*). The *p* value was determined using Wilcoxon rank-sum test. **e** First principal component of chr18, representing the open A-type (*black*) and closed B-type (*grey*) compartmentalization. *Highlighted bars* represent examples of regions with either stable or differential compartmentalization. The differential compartments are defined as genomic regions in which one type of compartmentalization is observed in one cell line and the other compartment type in the second cell line. **f** Pie chart showing the genomic compartment changes between MCF-10A and MCF-7 genomes. “*A*” and “*B*” denote the open and closed compartments, respectively. “*A → A*” represents compartments that are open in both cell lines, “*B → B*” represents compartments that are closed in both cell lines, “*A → B*” denotes compartments that are open in MCF-10A but closed in MCF-7, and “*B → A*” denotes compartments that are closed in MCF-10A and open in MCF-7. **g** Bar graph showing the percentage of compartments that have switched (A → B or B → A) or remained similar (A → A or B → B) between MCF-10A and MCF-7 genomes for chr16 through chr22 (*blue*) and the rest of the genome (*grey*). Chr16–22 display a higher percentage of B → A compartment switching, and a lower percentage of A → B compartment switching between MCF-10A and MCF-7, suggesting a more open compartmentalization in MCF-7. **P value < 0.001: Chi-square with Yates’ correction

Genome-wide interaction data were visualized as chromosome versus chromosome heat maps, where darker colors represent more frequent interaction events (Fig. 1a, b). The heat maps revealed two aspects of large-scale genome organization in the MCF-10A and MCF-7 cells. First, consistent with the notion of chromosome territories [11], intra*-*chromosomal interactions (visualized as darker boxes along the diagonal) were much more frequent than inter-chromosomal interactions (Fig. 1a, b). Second, we observed a number of large blocks of inter-chromosomal interactions representing the translocation events in these cell lines. Comparing the translocated regions in the Hi-C data with previously published MCF-10A and MCF-7 spectral karyotyping (SKY) and multiplex fluorescence in situ hybridization (M-FISH) data [49, 50], we observed that the majority of the translocated regions identified by SKY/M-FISH were also identified by Hi-C (Figures S3 and S4 in Additional file 1).

In order to assess whether the clustering of chromosomes is altered between MCF-10A and MCF-7 cells, we compared the genome-wide interaction differences (see "Materials and methods"; Fig. 1c). Strikingly, we observed a strong physical proximity of gene-rich, small chromosomes (chr16–22) in MCF-10A compared with MCF-7 (Fig. 1a–c, lower panels). This interaction network of small chromosomes also included the p-arm of chr8 (Fig. 1c). Quantification of the inter-chromosomal interactions between chr16 through chr22, and between chr16 through chr22 and the rest of the genome revealed that there is a significant increase of inter-chromosomal associations between chr16 through chr22 in the MCF-10A genome (Fig. 1d). The same result was also observed when, as an alternative approach, a direct subtraction of the MCF-10A and MCF-7 interaction matrices was performed (Figure S5a, b in Additional file 1). Moreover, the larger chromosomes (chr1–15 and X) in the MCF-10A genome showed similar levels of differential interaction frequency with other large chromosomes or chr16–22. Consistent with this observation, the positioning of chr18 with other small chromosomes was not prevalent in the raw Hi-C interaction matrices (Figure S6a–c in Additional file 1). However, the relative (MCF-10A/MCF-7) interaction frequency of chr18 with other small chromosomes was significantly increased in the MCF-10A cells (Figure S6d, e in Additional file 1), which suggests that all of the small chromosomes in MCF-10A cells show increased proximity to each other compared with the relative proximity in the MCF-7 cancer cell line.

### Decreased interaction frequency between small chromosomes in MCF-7 cells coincides with increased open chromatin compartmentalization

Previous evidence [21] has shown there are two unique patterns of interactions in the genome, representing the open (A-type) and closed (B-type) genomic compartments. We identified the two patterns of compartmentalization in both genomes with high reproducibility among the biological replicates (see "Materials and methods"; Figure. S7a, b in Additional file 1). Associating the MCF-7 ENCODE ChIP-seq datasets with the genomic compartments revealed the known features of genomic compartmentalization, including increased DNase I hypersensitivity, and higher levels of transcription factor binding in open (A-type) compartments in the MCF-7 genome (Figure S7c, d in Additional file 1).

To determine whether there are any differences in the compartmentalization between the MCF-10A and MCF-7 genomes, we compared the compartments throughout the genome at 250 kb resolution. The MCF-10A and MCF-7 genomes displayed similar distribution of open and closed compartments, with certain regions showing a change in genomic compartmentalization from A-type to B-type and vice versa (Fig. 1e, f). The majority of compartments were the same in both cell lines, where 47 % of all compartments constituted the A-type compartments and 40 % constituted the B-type compartments (Fig. 1f). Compartment switching was homogeneous throughout the chromosomes, rather than in a few hot spots (Figure S7e in Additional file 1).

Importantly, 12 % of all compartments in the MCF-10A genome transitioned to the opposite compartment (A-type to B-type and vice versa) in MCF-7 cells (Fig. 1f). To understand if the inter-chromosomal interaction changes we observed between small chromosomes were related to any compartment change, we asked whether there was an enrichment in transition of genomic compartments on small chromosomes (chr16–22). We found a significant enrichment of genomic regions on chr16–22 that switched to the A-type compartment in MCF-7 cells from the B-type compartment in MCF-10A cells (Fig. 1g). Conversely, we also observed a significant decrease of compartment transition from A-type in MCF-10A to B-type in MCF-7 on small chromosomes (Fig. 1g). These findings show that there is a higher frequency of open compartments on small chromosomes in the MCF-7 genome, which suggests a relationship between inter-chromosomal clustering, compartmentalization and phenotypic gene expression.

### Decreased inter-chromosomal interactions and higher frequency of open compartmentalization on chr16–22 in MCF-7 cells are associated with WNT signaling-related genes

**Fig. 2:**
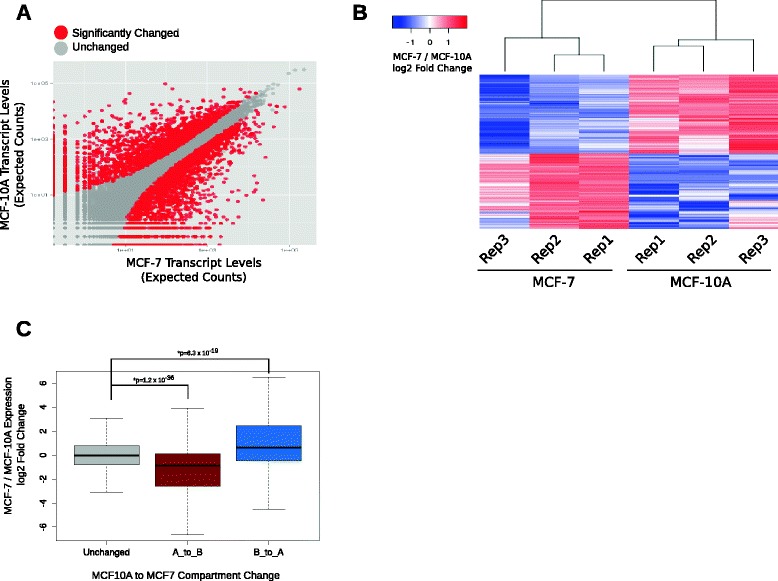
Differentially expressed genes are enriched at cell-specific genomic compartments. **a** Scatter plot showing differential gene expression between MCF-10A and MCF-7 cells. The axes represent normalized RNA-seq log2 gene expression counts. *Red dots* denote genes whose expression changed significantly and *grey dots* denote genes whose expression was unchanged. **b** Heatmap showing the MCF-7 up- and down-regulated genes for each biological replicate. Differential expression analyses identified 2437 MCF-7 up-regulated and 2427 MCF-7 down-regulated genes (log2 fold change > 1, *p* < 0.01) with high reproducibility. **c** MCF-7/MCF-10A log2 fold change expression boxplot of all the genes residing at regions for different compartmental switch categories. The compartments that are A → B and B → A show significantly decreased and increased expression levels, respectively. The *p* valuewas determined with Wilcoxon rank-sum test

Open compartmentalization is correlated with increased gene expression. We asked if the differential interaction network and compartmentalization of chr16 through chr22 between MCF-10A and MCF-7 cells are associated with differential gene expression. First, to characterize the gene expression differences between MCF-10A and MCF-7 cells, we performed RNA-seq with ribosomal RNA-depleted RNA from MCF-10A and MCF-7 cells with biological triplicates (Figure S8a, b in Additional file 1). Differential expression analyses identified 2437 MCF-7 up-regulated and 2427 MCF-7 down-regulated genes (log2 fold change > 1, *p* < 0.01) with high reproducibility (Fig. 2a, b). The number of differentially expressed genes identified in this study is comparable to previously published microarray studies [51]. The significant expression changes were enriched for the medium to highly expressed genes (Figure S8c in Additional file 1). The gene ontology terms associated with MCF-7 down-regulated (i.e., MCF-10A over-expressed) genes included terms such as “hemidesmosome assembly”, “focal adhesion”, and “neutral lipid biosynthetic process” (Additional file 2). On the other hand, gene ontology terms associated with MCF-7 up-regulated genes included terms such as “calcium-dependent cell adhesion” (Additional file 2).

To test the link between genome-wide open spatial compartmentalization and increased gene expression more directly, we analyzed the frequency of differentially expressed genes at regions where a compartment transition is observed. In agreement with previous findings [52], MCF-7 down-regulated genes were enriched in regions where the open A-type compartment in MCF-10A transitioned to a closed B-type compartment in MCF-7 (Fig. 2c). Conversely, there was an enrichment of MCF-7 up-regulated genes in regions with a B-type compartment in MCF-10A that switched to an A-type compartment in MCF-7 (Fig. 2c). In other words, when the MCF-7/MCF-10A log2 fold change expression levels were plotted for each compartment change category, we observed a down-regulation of MCF-7 genes in A-type to B-type compartment switch regions and an up-regulation of MCF-7 genes in B-type to A-type switch regions, respectively (Fig. 2c). These results show that compartment changes in the genome reflect differential gene expression.

Finally, to assess whether the differences in interactions and genomic compartments among the small chromosomes are associated with altered gene expression, we focused on the MCF-7 up-regulated genes on small chromosomes where the compartmentalization was switched from B-type to A-type (MCF-10A to MCF-7). REACTOME pathway analysis of these genes revealed well known oncogenic pathways, including “repression of WNT target genes” and “TCF/LEF binding to gene promoters” (Additional file 3).

Taken together, these results suggest that the decrease of inter-chromosomal associations of small chromosomes in the MCF-7 genome is associated with a higher open compartmentalization in MCF-7 and expression of genes related to the WNT signaling pathway, which is frequently implicated in tumorigenesis.

### Cell-line specific TAD boundaries are conserved between MCF-10A and MCF-7

**Fig. 3:**
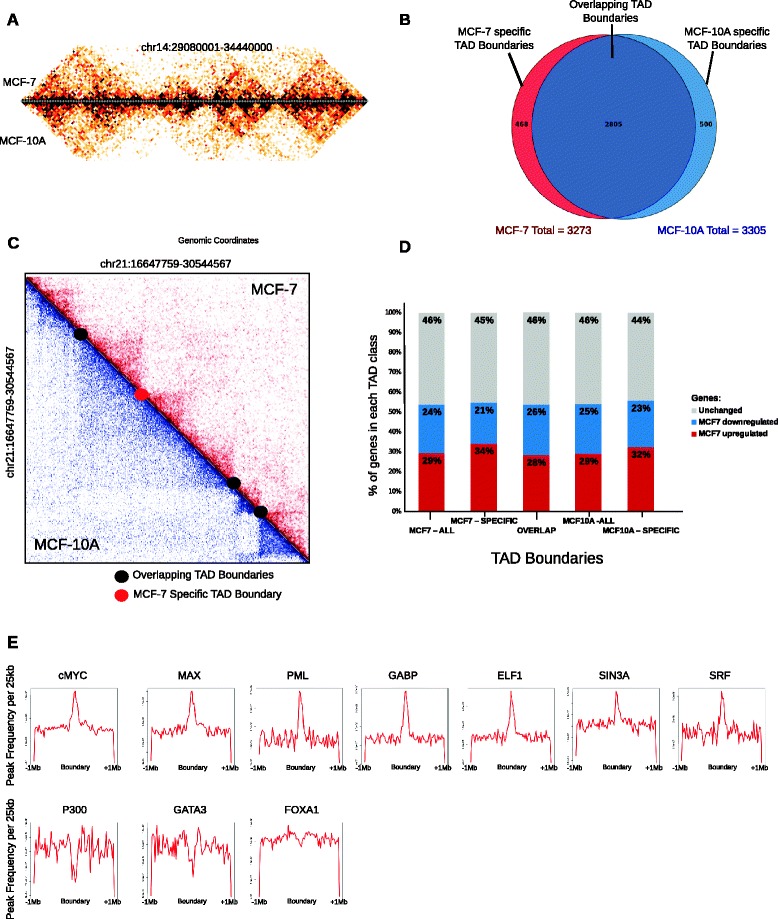
Topologically associating domains are similar between MCF-10A and MCF-7. **a** TADs are similar between MCF-10A and MCF-7 genomes. An example heatmap of a portion of MCF-10A chr14 at 40 kb resolution, where the upper part of the heatmap shows the MCF-7 TADs and the bottom part shows the MCF-10A TADs. **b** Venn diagram showing that the majority (~85 %) of all the TAD boundaries between MCF7 and MCF10A are conserved. **c** Heatmap showing an example of a differential TAD between MCF-10A (*blue*) and MCF-7 (*red*) on chr21 (chr21:16647759–30544567). The *black dots* represent the overlapping boundaries that are present in both cell lines, and the *red dot* denotes the MCF7-specific TAD boundary. **d** The percentage of unchanged (*grey*), MCF7 down-regulated (*blue*) and MCF7 up-regulated (*red*) genes located at each TAD boundary category. **e** Frequency plots of factors enriched at MCF- 7 TAD boundaries per 25 kb for ±1 Mb of every MCF-7 TAD boundary

Chromosome conformation capture-based studies revealed that A-type and B-type compartments are composed of TADs, where the expression levels of the genes in a single TAD can be co-regulated [24, 28, 53]. TADs have been shown to be stable units in different species, cell types, and physiological conditions [24, 28]. However, whether the large-scale chromosomal interactions and altered genomic compartments observed between MCF-10A and MCF-7 genomes have an effect on the structure of the underlying TAD formation and ultimately on gene expression is unknown. To address this question, we identified the TAD boundaries by calculating the insulation plot of the 40 kb resolution genome-wide interaction maps (see "Materials and methods"; Figure S9a in Additional file 1), with high reproducibility between the biological replicates (Figure S9b in Additional file 1). We detected 3305 and 3272 TAD boundaries in MCF-10A and MCF-7 genomes, respectively. Despite the differences in chromosomal structure and changes in compartmentalization and gene expression, ~85 % (2805) of the TAD boundaries were common between the cell lines (Fig. 3a, b). This rate of TAD boundary overlap is consistent with previous comparisons in different cell types and conditions [24, 28]. This result suggests that despite having cell type-specific translocations and large-scale structural differences, TAD boundaries are consistent between non-tumorigenic and tumorigenic cells.

Closer examination of TAD boundaries revealed that several TADs were “broken” into multiple sub-TADs between the cell lines. The boundaries that were shared among the larger and smaller TADs between the cell lines were categorized as “overlapping”, and the boundaries that were unique to a cell line were categorized as “cell line-specific” boundaries (Fig. 3c). We asked whether the genes residing at the cell line-specific boundaries showed cell line-specific differential gene expression. When the percentages of unchanged and MCF-7 up- and down-regulated genes were plotted per TAD boundary category, we did not find a strong correlation between cell type-specific TAD boundaries and differential gene expression (Fig. 3d).

As well as the TAD boundaries, we also analyzed the TADs. We categorized the TADs as overlapping (>90 % overlap), MCF-7-specific or MCF-10A-specific (see "Materials and methods" and below) (Figure S10a in Additional file 1). The overlapping TADs were slightly larger in size than the cell line-specific TADs (Figure S10b in Additional file 1). We then asked whether cell line-specific TADs showed differential gene expression. Analysis of differential gene expression for each TAD category showed that cell type-specificity of the TADs was not correlated with cell type-specific gene expression (Figure S10c in Additional file 1).

### MCF-7 TAD boundaries are enriched for several oncoproteins

TAD boundaries are bound by multiple factors [24, 54]. To investigate the chromatin states of the boundaries, we calculated the enrichment of factors characterized by MCF-7 ENCODE datasets at the MCF-7 TAD boundaries (Fig. 3e; Figure S10d in Additional file 1). The known features of the TAD boundaries, such as the enrichment of H3K36me3, CTCF, RAD21, transcription start sites, POL2, and DNase I hypersensitive sites, and the depletion of H3K9me3, were observed at the MCF-7 TAD boundaries (Figure S10d in Additional file 1). Interestingly, we observed a strong association of GABP, ELF1, PML, SIN3A, SRF, and the oncogenic drivers cMYC and MAX at MCF-7 TAD boundaries, and a depletion of GATA3 and FOXA1 (Fig. 3e). Consistent with previous work [24], P300 was depleted at the MCF-7 boundary regions. The rest of the MCF-7 ENCODE datasets did not show any enrichment (data not shown).

Recent evidence suggested that TADs may act as stable units of replication domains [55]. Therefore, we intersected the previously published MCF-7 Repli-seq dataset [55] with MCF-7 TAD boundaries and, consistent with the literature, we determined that late replicating regions were depleted at TAD boundary regions (Figure S11a in Additional file 1). Moreover, expression quantitative trait loci (eQTLs) have been shown to be preferentially located at TAD boundaries [56]. Integrating the breast cancer eQTL data [57] with MCF-7 TAD boundaries, we determined that breast cancer-associated eQTLs were enriched in overlapping TAD boundaries (Figure S11b in Additional file 1). Altogether, these results uncover previously unidentified transcription factors and chromatin states that may potentially play roles at the TAD boundaries.

### The telomeric/sub-telomeric regions in the MCF-10A genome display stronger associations than those in the MCF-7 genome

**Fig. 4:**
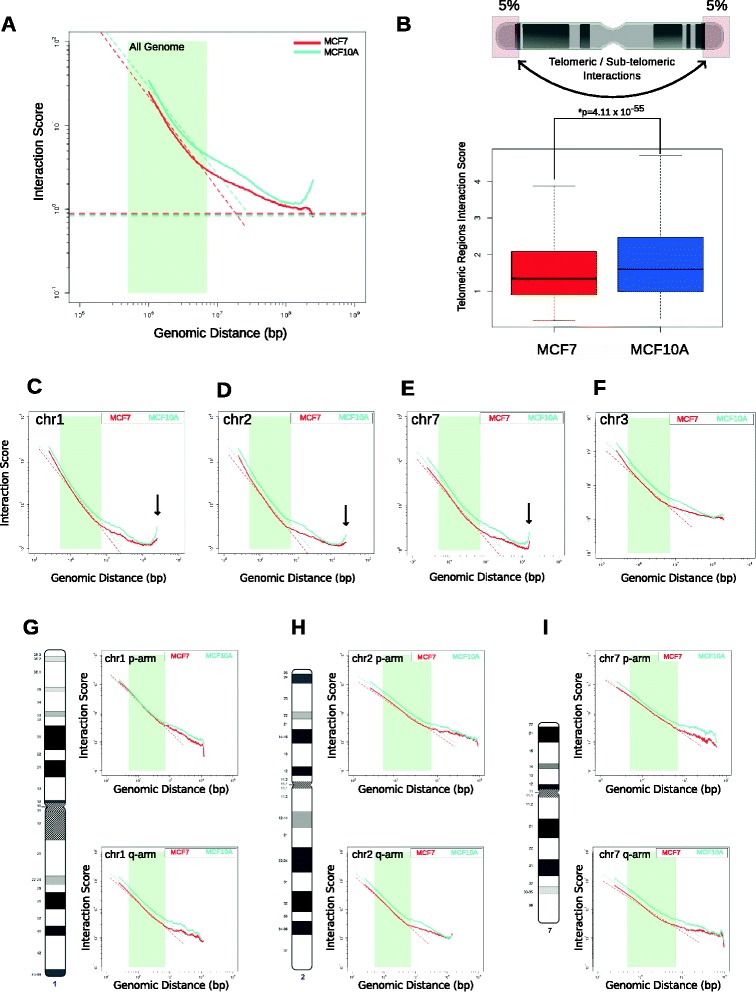
Telomeric and sub-telomeric regions in the MCF-10A genome display increased interaction frequencies. **a** Scaling plot of interaction frequencies against genomic distance for the MCF7 and MCF10A genomes. The MCF10A genome showed increased interaction frequency at genomic distances >200 Mb, suggesting telomere/sub-telomere associations. **b** Quantification of the interaction frequency between the telomeric regions (5 % of the ends by length) of each chromosome in MCF7 and MCF10A. The *p* value was determined by Wilcoxon rank-sum test. Scaling plots of MCF-10A and MCF-7 for chr1 (**c**), chr2 (**d**), chr7 (**e**), and chr3 (**f**). Chromosomes 1, 2 and 7 displayed an increased interaction frequency at large distances in MCF-10A but chromosome 3 did not. Scaling plots of individual chromosome arms for chr1 (**g**), chr2 (**h**), and chr7 (**i**)

Previous evidence has shown that interaction frequency decreases as a function of genomic distance [21]. This phenomenon represents the nature of the chromatin fiber and is a reflection of the folding status of the underlying chromatin [58]. We first asked whether the fiber characteristics of the MCF-10A and MCF-7 genomes were similar. Scaling plots of 1 Mb binned genome-wide intra-chromosomal interactions displayed the expected exponential decrease of contact probability as a function of increasing genomic distance in both MCF-10A and MCF-7 cells (Fig. 4a). Surprisingly, and in contrast to all previously published human Hi-C datasets, the frequency of interactions in MCF-10A showed an increase at very large genomic distances (>200 Mb; Fig. 4a). This suggests that very distant (i.e., telomeric/sub-telomeric) regions of chromosomes show a higher interaction frequency on the same chromosome. To assess whether the telomeric ends of the chromosomes in MCF-10A indeed have higher frequencies of interactions compared with those in MCF-7, we calculated the intra-chromosomal interaction frequency of the ends of each chromosome (5 % by length) in MCF-10A and MCF-7 cells. We observed a significant increase in telomeric/sub-telomeric interaction frequency in the MCF-10A genome (Fig. 4b), which supports the observation that intra-chromosomal telomeric interactions are more frequent in MCF-10A cells. Scaling plots of each chromosome individually at 250 kb resolution indicate that the increase in telomeric/sub-telomeric interactions seemed to be driven by chr1, chr2, and chr7 in the MCF-10A genome (Fig. 4c–e; Figure S12 in Additional file 1). However, this phenomenon was not observed on other large chromosomes in MCF-10A cells, such as chr3 (Fig. 4f; Figure S12 in Additional file 1). Certain chromosomes, such as chr11 and chr16, showed increased interaction frequency at large distances in both the MCF-10A and MCF-7 genomes even though their lengths did not span 200 Mb (Figure S12 in Additional file 1). As expected, this was not observed when the scaling plots for individual chromosomal arms were analyzed (Fig. 4g–i; Figure S13 in Additional file 1).

These results suggest that the telomeric ends of the chromosomes, especially chr1, chr2, and chr7, in the MCF-10A genome are in closer proximity than those in MCF-7. Taken together, we identified large-scale differences in both *cis-* and *trans-*chromosomal interactions between two commonly used cell lines in breast cancer research.

## Discussion

Cancer is a disease characterized by major morphological changes in the nucleus [8, 9]. Although individual gene positioning may differ [16], the relative arrangement of chromosomes in the interphase nucleus can be conserved between normal and cancer cells [59]. Furthermore, extensive epigenetic dysregulation is observed in the cancer state. In order to map the genome-wide interactions and perform a comparative analysis, we performed Hi-C in the MCF-10A and MCF-7 cell lines. We observed a higher background interaction frequency in the MCF-7 genome compared with the MCF-10A genome (Fig. 1a, b). This background could be the result of a technical source (i.e., the ligation step in the Hi-C procedure) or because of increased background interaction frequency in the MCF-7 genome due to the probabilistic positioning of the chromosomes inside the aneuploid nucleus and increased diversity of interactions within this genome.

Comparison of MCF-7 and MCF-10A Hi-C data revealed a significant depletion of inter-chromosomal associations between small, gene-rich chromosomes (chr16–22) in the MCF-7 genome. One possibility for the loss of interactions among the small chromosomes in MCF-7 compared with MCF-10A cells is that randomization (i.e., loss of specificity) of contacts within the MCF-7 genome could lead to lower frequencies of individual contacts, and hence to an apparent loss of interaction. However, loss of specific contacts does not itself cause a difference in overall chromosome contacts. Two whole chromosomes that tend to be close together in a cell will overall show more inter-chromosomal interactions with each other by Hi-C than will two distant chromosomes, even if they have no specific interactions that are consistent across the population of cells. If each cell in the population has a different arrangement of chromosome territories, this will look, on average, like less clustering of small chromosomes. But this scenario should also reveal more interactions between large and small chromosomes and less clustering of large chromosomes. In Figure S5 in Additional file 1 and Fig. 1c, in contrast, we do not observe a compensating increase in interactions between the small and large chromosomes, suggesting that this is not just a randomization of interactions. Moreover, it should be kept in mind that there are several extensive rearrangements in the MCF-7 genome, and it could be that only the re-arranged copies of a highly aneuploid chromosome may show a particular three-dimensional conformation.

The decreased clustering of small chromosomes and the differentially open compartmentalized regions in MCF-7 are associated with increased expression of genes related to tumorigenesis. The correlation between increased gene expression in B-type to A-type compartment switch regions and a higher number of A-type compartments on chr16–22 in MCF-7 cells suggests that the underlying mechanism for this phenomenon is most likely due to transcriptional differences, rather than chromosomal copy number changes between the cell lines. The loss of small chromosome clustering may also be interpreted as a reflection of mis-organization of the chromosome territories in cancer.

Genomic compartmentalization has been shown to be associated with gene expression [21, 52]. One hypothesis for the clustering, compartmental, and transcriptional changes we observe in small chromosomes would be that once a gene is activated/repressed in the process of tumorigenesis, its position in the three-dimensional nuclear space is changed, with movement towards the open/closed compartment regions. Such a phenomenon has been previously shown by microscopic studies [60]. An alternative hypothesis is that chromosomes change compartments before gene expression changes. A recent study supports the alternative hypothesis in which chromatin decondensation plays a major role in cell differentiation [61].

Scaling plot analysis (Fig. 4) suggested that distinct types of chromatin folding states might exist between MCF-10A and MCF-7 cells, both genome-wide and at individual chromosomes [58]. Surprisingly, and in contrast to all previous human Hi-C datasets, there was an increased frequency of interactions at distances >200 Mb in MCF-10A cells, suggesting interactions between telomeric and sub-telomeric regions on the same chromosome. It has been suggested that telomere clustering is associated with the alternate lengthening of telomeres (ALT) mechanism [62]. ALT is a mechanism in which telomere length is maintained through a homologous recombination-dependent process. It could be possible that the MCF-10A and MCF-7 cells have different mechanisms of telomere maintenance, and the proximity of telomeric ends in the MCF-10A genome might suggest an effect of increased ALT regulation. Increased telomere interactions were observed in chr1, chr2, and chr7, and on some smaller chromosomes (Figure S12 in Additional file 1), but not in individual chromosomal arms (Figure S13 in Additional file 1). A recent report suggests that 10 % of all cancers and immortalized cell lines display the ALT mechanism [63]. Our results are consistent with previous findings that the presence of an ALT mechanism results in clustering of telomeres, which is observed in epithelial MCF-10A cells but not in tumorigenic MCF-7 cells.

Overall, in this study we charted the chromatin structure of mammary epithelial and breast cancer cells at different chromosomal scales, from large-scale chromosomal *cis*- and *trans*-interactions to genomic compartmentalization and TAD formation (Figure S14 in Additional file 1). Further studies on normal and cancer genomes and primary cells will provide additional insight into the functional role of chromatin organization in transcriptional regulation and tumorigenesis.

## Conclusions

This study provides a genome-wide molecular view of alterations in the three-dimensional chromatin organization between epithelial and breast cancer cells.

## Materials and methods

### Cell culture

MCF-10A cells were obtained from the Barbara Ann Karmanos Cancer Institute (Detroit, MI, USA). The cells were maintained in monolayer in Dulbecco’s modified Eagle’s medium-F12 (DMEM/F12; Invitrogen, 21041025) supplemented with 5 % horse serum (Invitrogen, 16050122), 1 % penicillin/streptomycin (Invitrogen, 15140122), 0.5 μg/ml hydrocortisone (Sigma, H-0888), 100 ng/ml cholera toxin (Sigma, C-8052), 10 μg/ml insulin (Sigma, I-1882), and 20 ng/ml recombinant human epidermal growth factor (Peprotech, 100–15) as previously described [64]. MCF-7 cells were obtained from ATCC and were cultured in DMEM supplemented with 10 % fetal bovine serum and penicillin/streptomycin.

### RNA-seq and analysis

The RNA-seq libraries were generated with TruSeq Stranded Total RNA with Ribo-Zero Gold Kit and the samples were sequenced as 100-bp single-end reads using a Hi-Seq 2000 instrument. For the RNA-Seq analysis, the adapter sequences were first removed from the RNA-seq reads. Ribosomal RNA reads, if any, were filtered out using Bowtie [65]. After quality filtering and adapter removal steps, the reads were aligned to a transcriptome and quantified using RSEM v.1.2.7 [66]. The annotation file was downloaded from University of California, Santa Cruz (UCSC) genome browser, human hg19 assembly. To quantify gene expression, gene counts and transcripts per million (TPM) were calculated by using the RSEM tool. Differential gene expression was calculated using the Deseq2 version 1.4.5 package in R 3.1.0 using the mean value of gene-wise dispersion estimates [67]. To find significant differentially expressed genes, we used 0.01 for adjusted *p* value and >1 log2 fold change. Gene ontology analysis was performed with the FuncAssociate software [68]. The RNA-seq plots were confirmed using the ngs.plot software [69].

### Preparation of Hi-C libraries

Hi-C was performed as previously described with minor modifications [46]. The modified part of the protocol was in the biotin incorporation step, where the mixture was incubated at 37 °C for 40 minutes with continuous shaking and tapping of the tube every 10 minutes. The MCF-10A and MCF-7 Hi-C samples displayed a range of 40–85 % biotin incorporation efficiency. At the end of Hi-C sample preparation, the libraries were sequenced using PE100 reads with a Hi-Seq 2000 instrument.

### Read mapping/binning/ICE correction

Figure S1 in Additional file 1 summarizes the mapping results and different classes of reads and interactions observed [47]. The data were binned at 6.5-Mb, 1-Mb, 250-kb, 100-kb, and 40-kb non-overlapping genomic intervals. In our Hi-C analyses of the near diploid MCF-10A and aneuploidy MCF-7 cells, we utilized the iterative correction and eigenvector decomposition (ICE) method [48], which corrects for differences in copy number. A tetraploid chromosome may have twice as many sequenced interactions as a diploid chromosome, but the ICE method divides its final interaction counts by the total sum of all interactions and thus normalizes this difference. Iterative mapping and correction of Hi-C data were performed as previously described [48]. Biological replicates showed high reproducibility (Pearson’s correlation coefficient >0.9 for 1 Mb resolution data). Similarly, the first eigenvector comparison of the replicates showed high reproducibility (Figure S7a in Additional file 1). For the downstream analyses, sequences obtained from both biological replicates were pooled and ICE-corrected to serve as a combined dataset.

### Z score calculation

We modeled the overall Hi-C decay with distance using a modified LOWESS method (alpha = 1 %, interquartile range filter), as described previously [70]. LOWESS calculates the weighted-average and weighted-standard deviation for every genomic distance and therefore normalizes for genomic distance signal bias.

### Calculation of differential interactions

To capture the differences between MCF-10A and MCF-7 interactions, we first transformed the 6.5-Mb Hi-C data into Z score matrices for all four replicate datasets (MCF-7-R1, MCF-7-R2, MCF-10A-R1, and MCF-10A-R2). For each interaction, the mean sample:sample (between samples) Z score difference was calculated from all pairwise combinations of the four datasets (MCF-7-R1 and MCF-10A-R1, MCF-7-R1 and MCF-10A-R2, MCF-7-R2 and MCF-10A-R1, MCF-7-R2 and MCF-10A-R2). The replicate:replicate Z score difference (within samples) was also calculated for a random set of 500,000 interactions. These random replicate–replicate Z score differences were then used to build an expected distribution of Z score differences. The resulting Z score difference matrix was then derived by calculating for each bin the ratio of the mean of the set of four possible sample:sample Z score differences minus the genome-wide mean of the replicate:replicate Z score difference, divided by the genome-wide standard error of the replicate:replicate Z score differences. For Figure S5 in Additional file 1, we performed a direct subtraction of the Z score matrices (MCF-7 minus MCF-10A).

### Compartment profiles

First, the Z scores of the interaction matrices at 250 kb resolution were generated as described previously [20]. Then, Pearson correlation on the Z score matrices was calculated. In performing principal component analysis [20, 21], the first principle component usually detects the patterns of increased and decreased interaction across the genome that appear as a “plaid pattern” in the heatmap. Each genomic region tends to match this prominent interaction pattern (positive eigenvector value) or its opposite (negative eigenvector value) and these represent the two spatially segregated compartments. In any given analysis, though, the generally open, gene-rich A-type compartment may end up with either a positive or negative eigenvector. To detect which compartment is the open A-type and which is the closed B-type, the genome-wide gene density was calculated to assign the A-type and B-type compartmentalization.

### Identification of TAD boundaries (insulation square analysis)

TAD calling was performed by calculating the “insulation” score of each bin using the 40 kb resolution combined Hi-C data. The mean of the interactions across each bin was calculated. By sliding a 1 Mb × 1 Mb (25 bins × 25 bins) square along the diagonal of the interaction matrix for every chromosome, we obtained the insulation score of the interaction matrix. Valleys in the insulation score indicate the depletion of Hi-C interactions occurring across a bin. These 40-kb valleys represent the TAD boundaries. Based on the variation of boundaries between replicates (Figure S9a in Additional file 1), we chose to add a total of 160 kb (80 kb to each side) to the boundary to account for replicate variation. The final boundaries span a 200-kb region. All boundaries with a boundary strength <0.15 were excluded as they were considered weak and non-reproducible. The insulation plots for the biological replicates showed high reproducibility (Pearson correlation coefficient = 0.80 for MCF-7 and 0.90 for MCF-10A replicates; Figure S9b in Additional file 1), suggesting the robustness of the method. Similarly, the overlap of detected boundaries also showed high reproducibility between the biological replicates (~85 % TAD boundary overlap for MCF-7 and ~91 % for MCF-10A). Therefore, we used the combined Hi-C replicates for the TAD analyses.

### Identification of TADs

The cell line-specific TADs were identified using the bedtools suite [71]. First the boundaries on all chromosomes for both MCF-10A and MCF-7 were merged. The boundaries that overlapped were categorized as “all overlapping TAD boundaries”. Then, the regions outside of the boundaries were extracted using the “complementBed” function. The telomere/centromere regions were filtered using the “intersectBed -v” option. The resulting regions constituted the “all overlapping TAD boundaries”. Next, the TAD boundaries identified in MCF-10A and MCF-7 datasets were independently subtracted (by using the subtractBed function) from the “all overlapping TAD boundaries”. Within these two independently subtracted datasets, the TADs that have at least 90 % overlap (−f 0.90 − r) were considered as “overlapping TADs”, TADs that were found only in MCF-7 were categorized as “MCF-7-specific TADs”, and the domains that were only found in MCF-10A subtracted datasets were categorized as “MCF-10A-specific TADs”.

### Availability of supporting data

The raw and processed RNA-seq and Hi-C datasets have been submitted to NCBI Gene Expression Omnibus (GEO) under accession numbers [GEO:GSE71862 and GSE66733].

## Additional files

Additional file 1:
**Supplementary Figure S1 to Figure S14.** (PDF 23309 kb) Additional file 2:
**A table listing the gene ontology terms of differentially expressed genes between MCF-10A and MCF-7 cells.** (XLSX 8 kb) Additional file 3:
**A table listing the gene ontology terms of the MCF-7 up-regulated genes that are on small chromosomes and are on compartment switch regions.** (XLSX 5 kb)

## Abbreviations

ALT: alternate lengthening of telomeres
Chr: chromosome
DMEM: Dulbecco’s modified Eagle’s medium
eQTL: expression quantitative trait locus
Hi-C: genome-wide chromosome conformation capture
ICE: iterative correction method
M-FISH: multiplex fluorescence in situ hybridization
SKY: spectral karyotyping
TAD: topologically associating domain
