# Supplementary material for: Chromatin interaction analysis reveals changes in small chromosome and telomere clustering between epithelial and breast cancer cells
Source: Genome Biol. 2015 Sep 28;16:214. doi: 10.1186/s13059-015-0768-0 (PMC4587679; doi:10.1186/s13059-015-0768-0)
Supplement: Additional file 1: — Supplementary Figure S1 to Figure S14. (PDF 23309 kb) [file 13059_2015_768_MOESM1_ESM.pdf]

Figure S1

a) Iterative Mapping Statistics

Iterat ve Mapping Stat st cs	side1TotalReads	side1ZeroAlignedReads	side1Mult pleAlignedReads	side1OneAlignedReads	side2TotalReads	side2ZeroAlignedReads	side2Mult pleAlignedReads	side2OneAlignedReads
**MCF10a-WT-Rep1**	159,334,130	33,272,095	3,830,572	122,231,463	159,334,130	35,079,522	3,708,345	120,546,263
**MCF10a-WT-Rep2**	130,640,941	19,171,967	4,112,221	107,356,753	130,640,941	20,653,438	3,967,826	106,019,677
**MCF7-WT-Rep1**	156,841,886	35,140,925	3,957,593	117,743,368	156,841,886	36,420,678	3,873,238	116,547,970
**MCF7-WT-Rep2**	129,444,410	19,465,398	3,749,200	106,229,812	129,444,410	20,566,318	3,660,077	105,218,015

b) HiC Library Statistics

HiC Library Stat st cs	bothSideMapped	sameFrag	danglingEnds	selfCircles	errorPairs
**MCF10a-WT-Rep1**	88,760,876	6,350,116	2,063,283	4,260,751	25,814
**MCF10a-WT-Rep2**	86,597,914	8,674,320	1,423,166	7,104,270	143,550
**MCF7-WT-Rep1**	87,377,903	18,359,142	7,663,547	10,677,933	16,775
**MCF7-WT-Rep2**	85,679,799	10,389,096	2,509,374	7,846,506	28,801

c) HiC Interaction Filtering Statistics

HiC Interact on Filtering Stats	InteractionsBeforeRSiteFilter	InteractionsAfterRSiteFilter	InteractionsBeforeDupSiteFilter	InteractionsAfterDupSiteFilter	uniqueValidPair
**MCF10a-WT-Rep1**	82,410,759	82,252,534	82,252,534	74,800,826	**74,800,826**
**MCF10a-WT-Rep2**	77,923,587	77,543,097	77,543,097	76,727,093	**76,727,093**
**MCF7-WT-Rep1**	69,018,758	68,801,978	68,801,978	68,133,012	**68,133,012**
**MCF7-WT-Rep2**	75,290,693	75,094,614	75,094,614	74,545,436	**74,545,436**

Figure S1- Hi-C Library Sequencing Statistics

The sequencing, mapping and filtering statistics for the Hi-C replicates analyzed as previously described (Lajoie et al. 2015)

Figure S2

A)

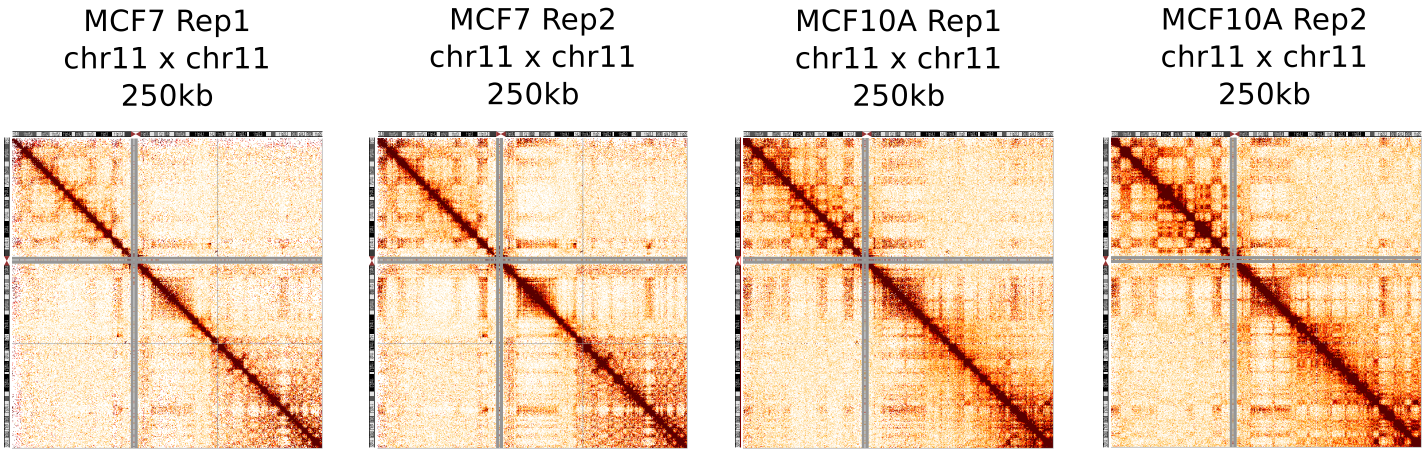

B)

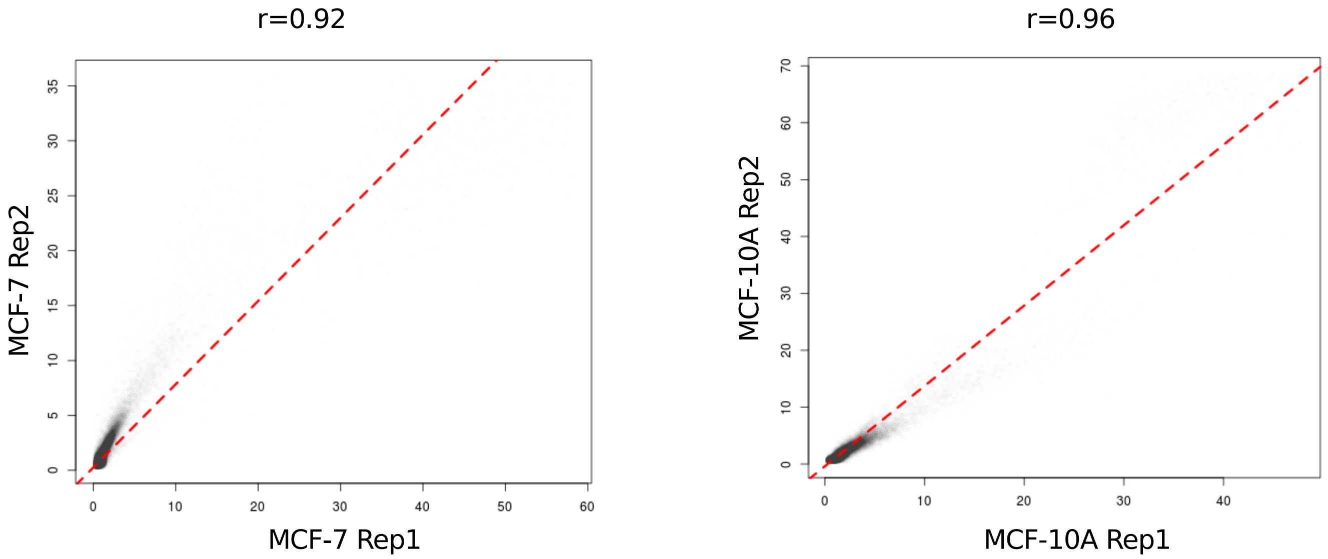

Figure S2- Example heatmaps and reproducibility of Hi-C replicates

A) Images 250kb interaction matrices of chr11 for each biological replicate

B) Pearson correlation coefficient of genome-wide interactions of biological replicates for each cell line

Figure S3

Cell line	No. of Chromosomes	No. of Markers	Markers
MCF10A	47	4	t(3;9), t(6;19), t(5;3;9) +(8)

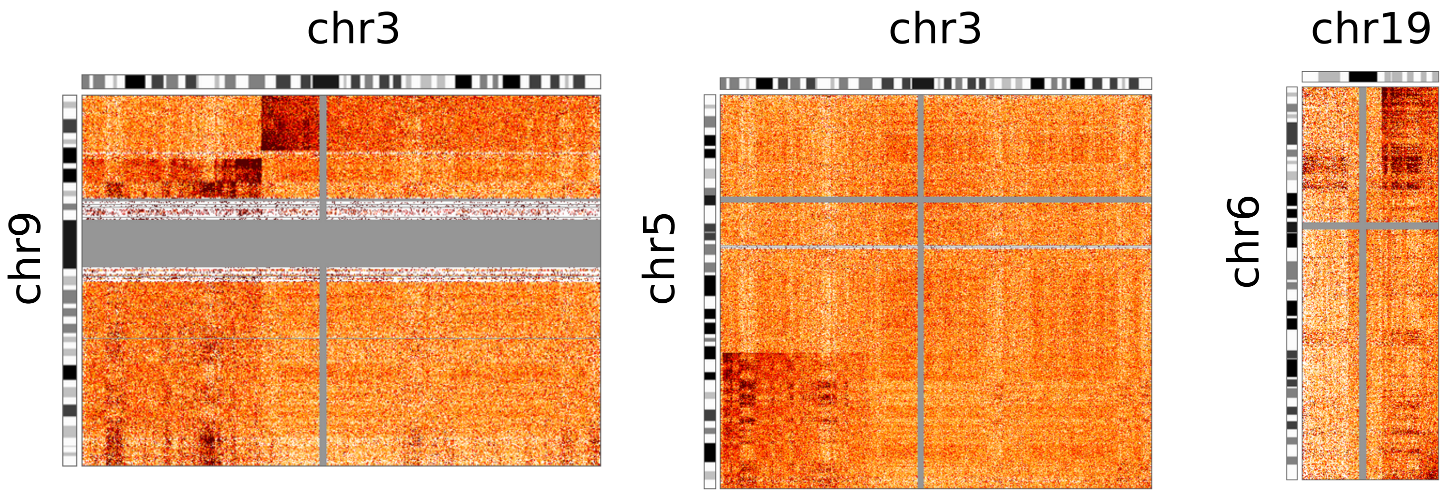

Figure S3 – Inter-chromosomal interaction heatmaps showing MCF-10A translocations

Inter-chromosomal heatmaps showing the translocations detected by a previous SKY-FISH study [49].

Figure S4

1x1	der(1)t(X;1)			
2x2	der(2)t(2;3)(q36;?)			
3x2	del(3)(p?)	der(3)t(3;6)		
4x3				
5x3	der(5)t(5;13)(p12?;q22)			
6x1	der(6)t(6;7)	der(6)t(3;6)		
7x1	der(7)t(1;19;7;6)	der(7)t(7;19;7)		
8x1	der(?)t(16;11;8;11;3)	der(8)t(8;15)	der(8?)t(8;12)	der(8)t(8;16)
9x2	der(?)t(6;20;9;3)	der(9)t(8;9)		
10x2	der(10)t(7;10)(?;q22)			
11x2	der(?)t(11;1;17;19;17)			
12x3	der(19)t(12;19)			
del(13)(q?)	der(13)t(13;15;11;16)	der(13)t(13;14)	der(13)t(13;16)	
14x2	der(?)t(7;14)			
15x2	der(16)t(15;16)			
16x2				
17x1	der(17)t(3;17)			
18x2	der(18)t(18;21)			
19x1	der(19)t(7;19)			
20x1	der(?)t(17;1;19;17;20)	der(20)t(3;20;1;20;1;	der(20)t(20;1;21?)	
21x3				
der(22)t(7;22)	der(22)t(16;22)			
Xx2 cp[6]				

Figure S4 – Inter-chromosomal interaction heatmaps showing MCF-7 translocations.
Inter-chromosomal heatmaps showing the translocations detected by a previous SKY-FISH study [48].

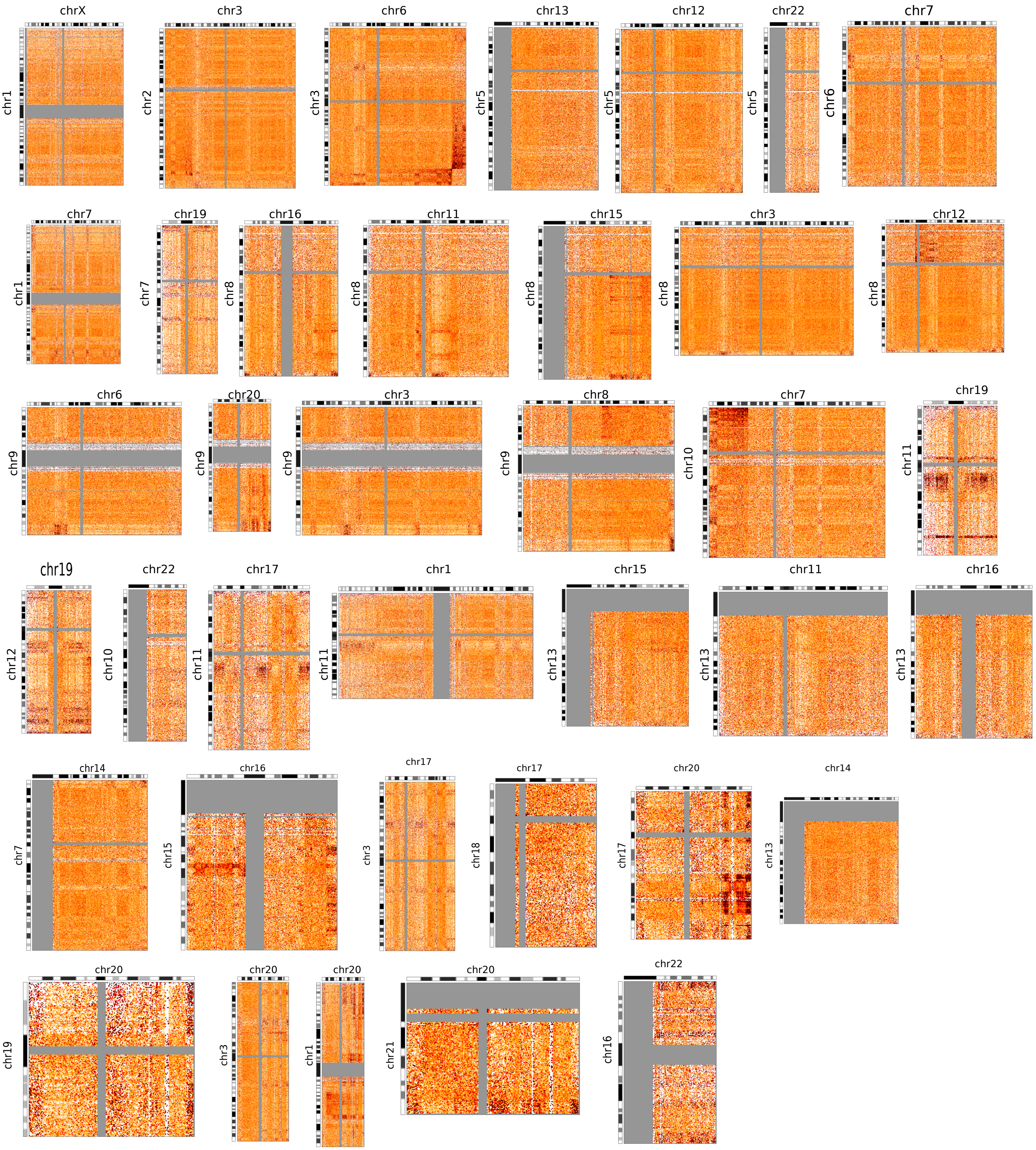

Figure S5

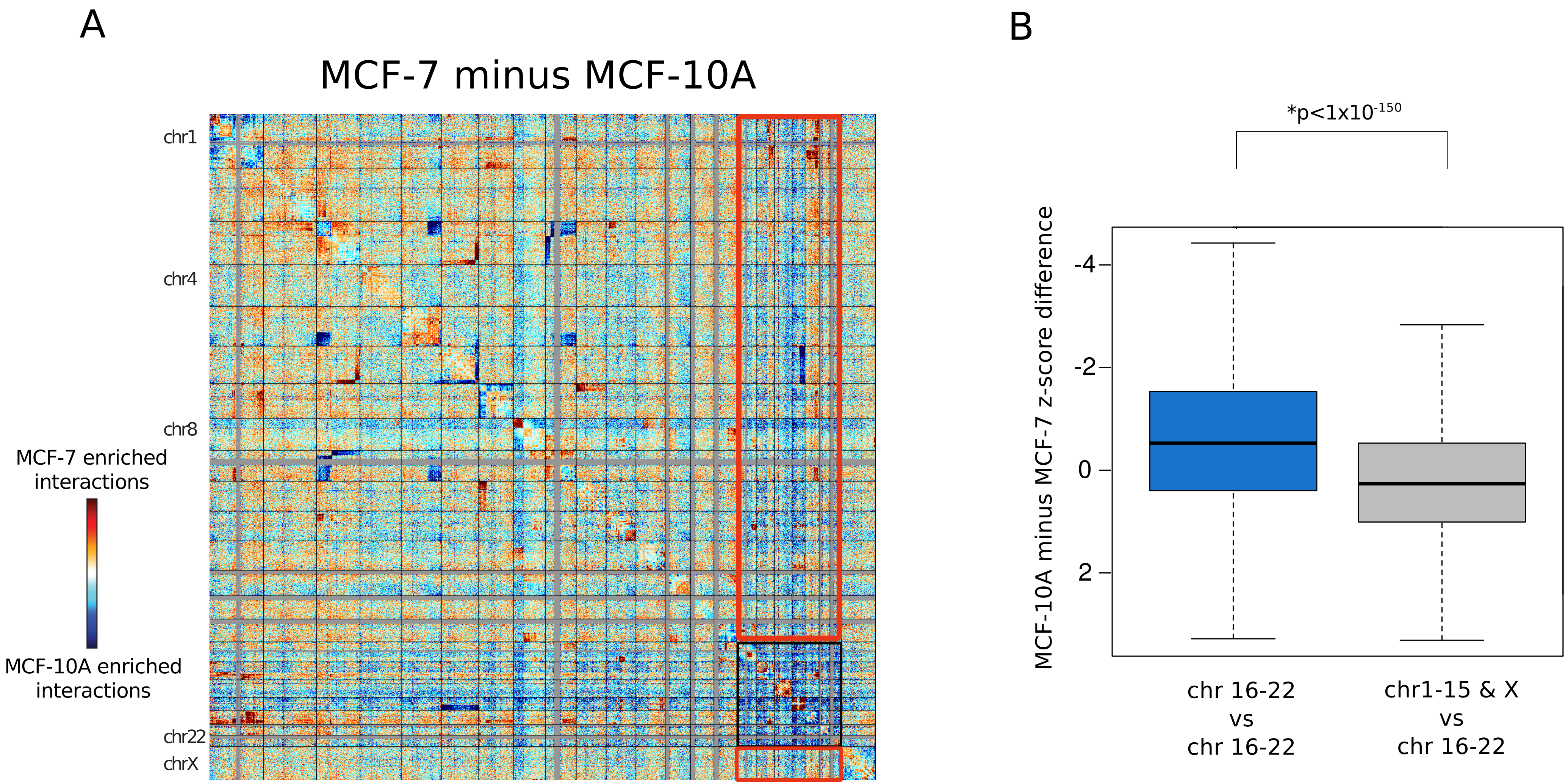

Figure S5 - Direct subtraction of heatmaps reveals that small chromosomes are more associated with each other in the MCF-10A genome

A. Subtraction of the MCF-10A z-score matrix from that of MCF-7 at 2.5Mb resolution. The blue and the red squares represent interactions enriched in the MCF-10A and MCF-7 genomes, respectively. The black rectangle denotes the interactions among small chromosomes (chr16-22) and the red rectangles show the interactions between the small chromosomes and chr1-15 and chrX.

B. Boxplot showing the quantification of interactions among chr16-22 (blue), or with rest of the genome (grey).p-value: Wilcoxon rank-sum test

Figure S6

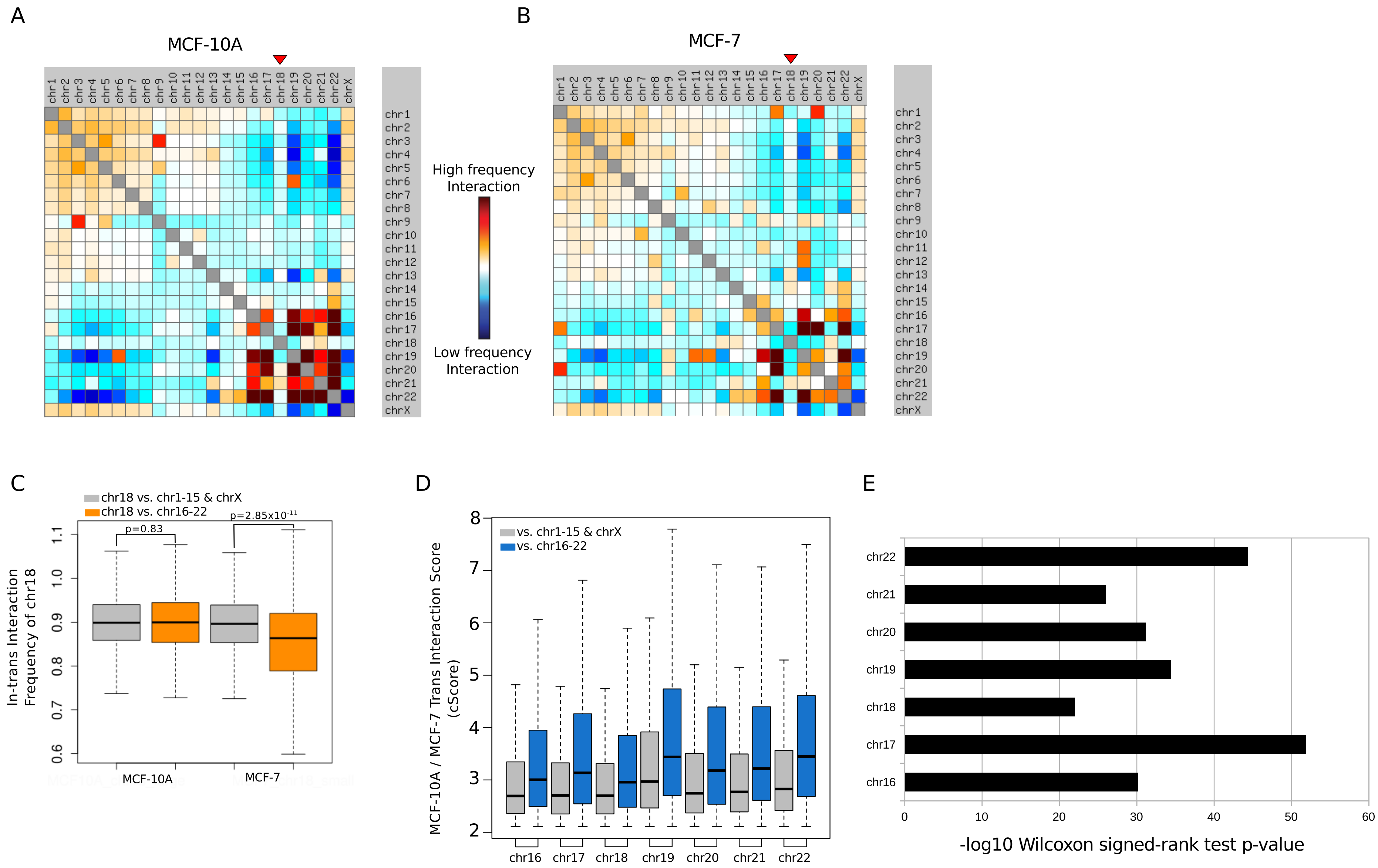

Figure S6. Analysis of inter-chromosomal interactions of small chromosomes

All by all raw interaction matrix for **A)** MCF-10A and **B)** MCF-7 genomes. The red arrow denotes chr18.
C) Interaction frequency of chr18 either with large chromosomes (grey), or the small chromosomes (orange) in MCF-10A and MCF-7 cells. The MCF-7 chr18 displays less interaction frequency with other small chromosomes compared to MCF-10A chr18.
D) Boxplots showing the relative interaction frequency (MCF-10A / MCF-7) of each small chromosome. **E)** p-values of the differences between small and large chromosome clustering of each small chromosome in Panel D, assessed by Wilcoxon rank-sum test

Figure S7

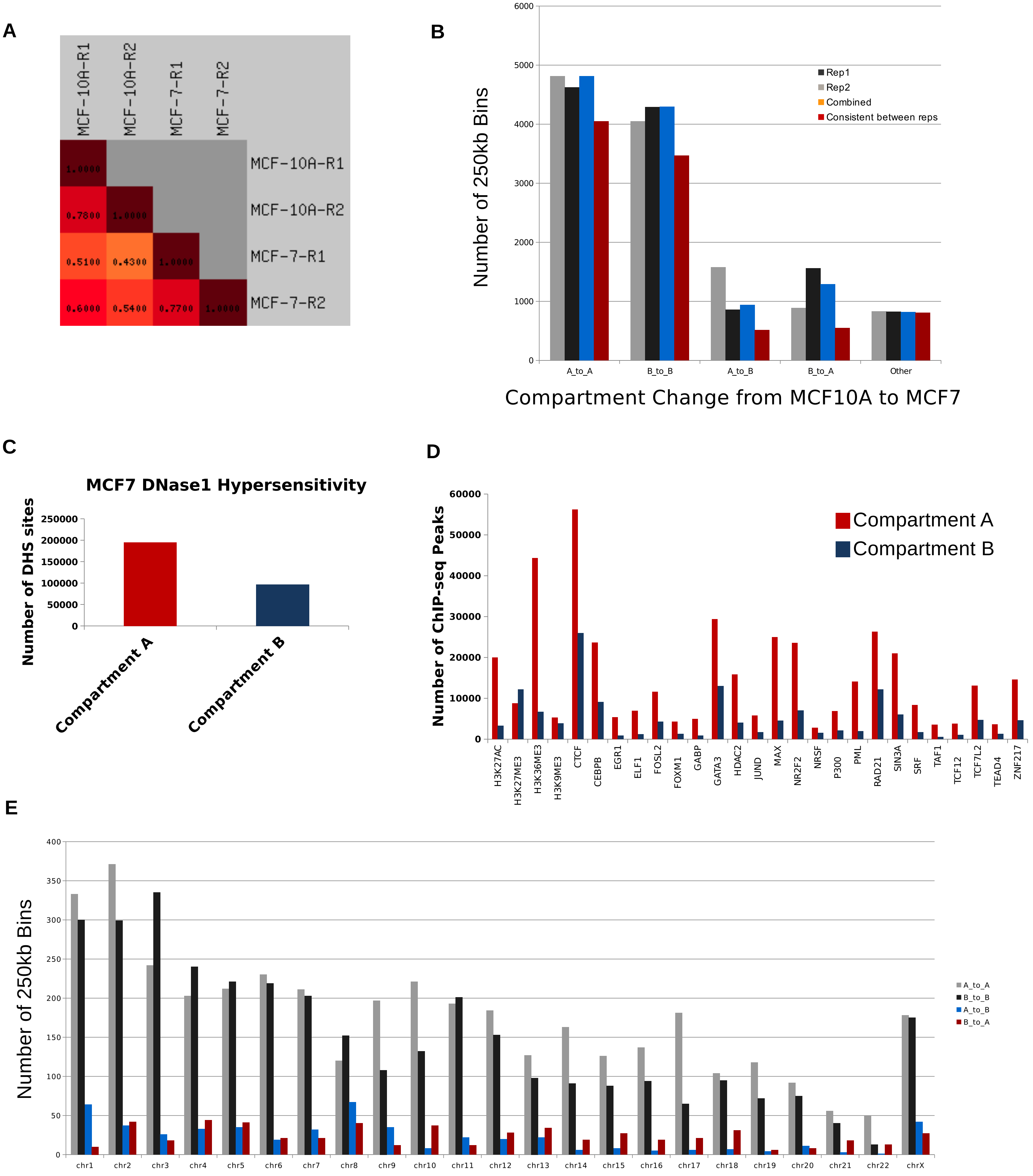

Figure S7- Compartments are reproducible

A) Pearson correlations of the 1st eigen values for each Hi-C biological replicate
B) Bar-graph showing the number of reproducible compartmentalization between the replicates. "Other" indicates repetative regions that are masked. Only the compartments that are consistent between the compartments are used for downstream analyses.
C) Number of DNase1 hypersensitive sites in MCF-7 in open or closed compartments
D) Number of ENCODE transcription factor ChIP-seq peaks per compartment in MCF-7
E) Bargraph displaying the A-type to B-type and vice versa compartment changes for each chromosome. The compartment switching is distributed throughout each chromosome.

Figure S8

A MCF--10A Replicates Transcript Per Million (TPM) Pearson Correlation R^2 Values

	Replicate1	Replicate2	Replicate3
Replicate1			
Replicate2	0.94		
Replicate3	0.97	0.93	

B MCF--7 Replicates Transcript Per Million (TPM) Pearson Correlation R^2 Values

	Replicate1	Replicate2	Replicate3
Replicate1			
Replicate2	0.99		
Replicate3	0.99	0.99	

C

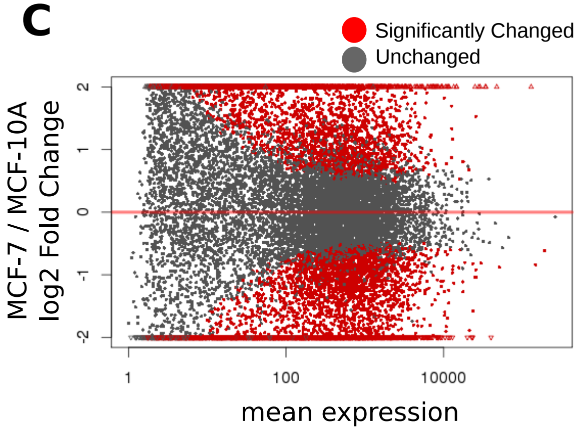

Figure S8- RNA-seq replicate correlation

The pearson correlations of the transcript per million (TPM) values for each biological replicate of A) MCF-10A and B) MCF-7 RNAseq data.
C) MA-plot showing the log2 fold change versus the mean expression value of significantly differentially expressed genes

Figure S9

A

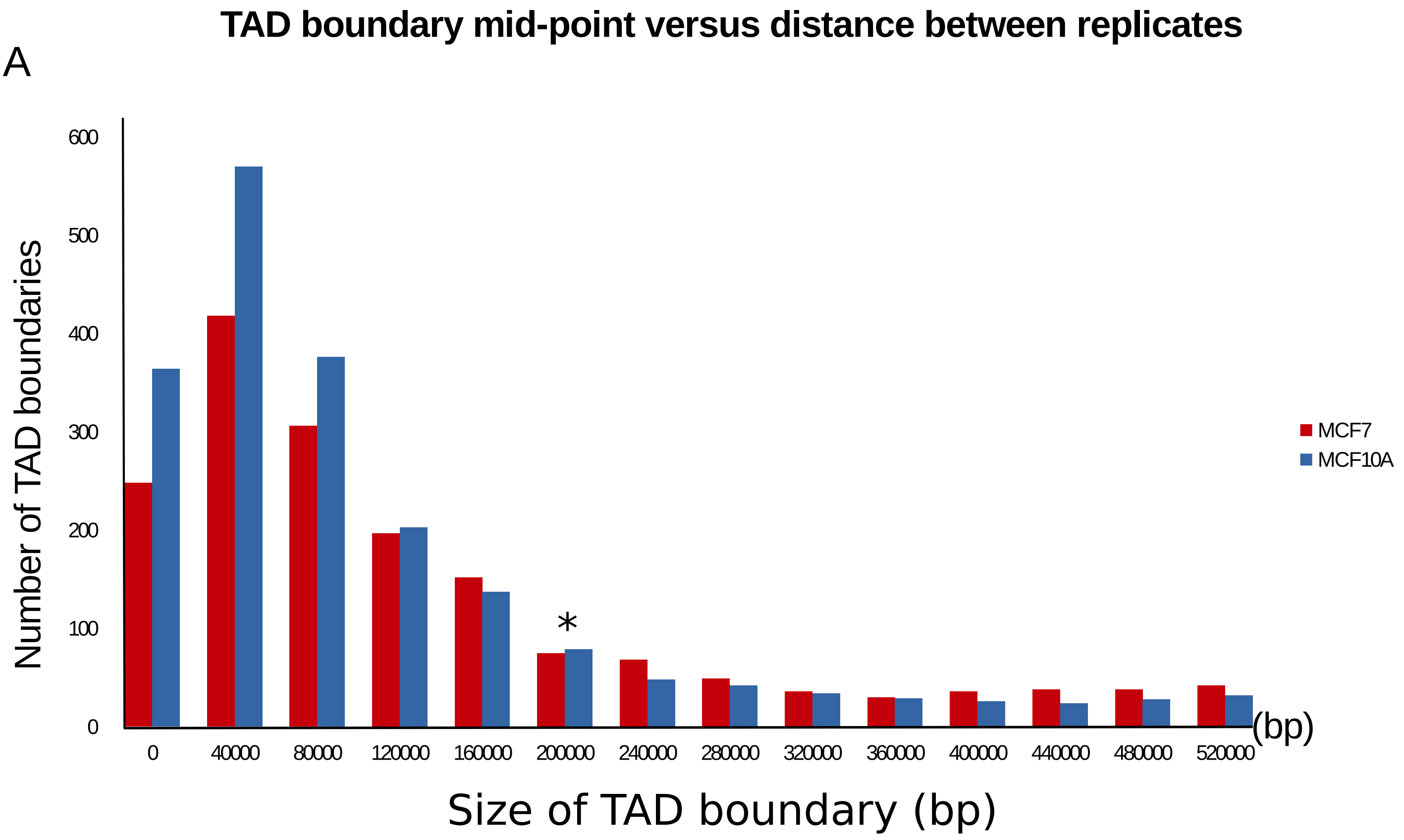

B

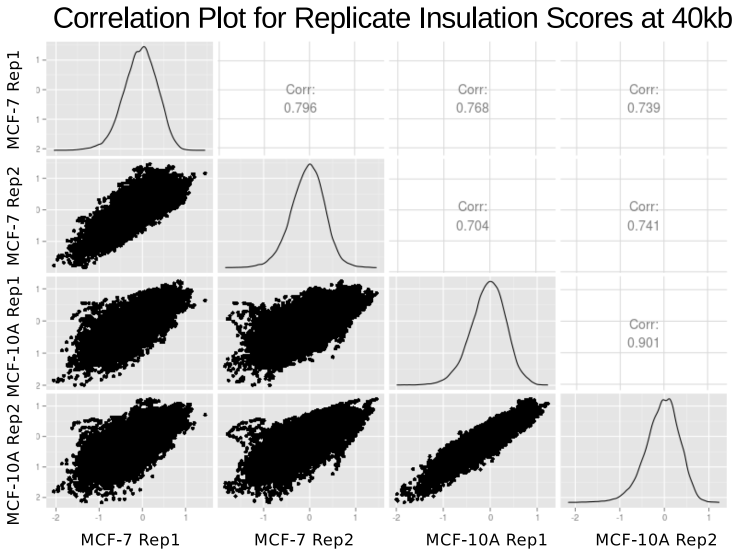

Figure S9- TAD boundary analyses
A) Graph showing the number of shared TAD boundaries in relation to their set size (see Methods)

B) Correlations for Insulation Plot at 40kb for each biological replicate

Figure S10

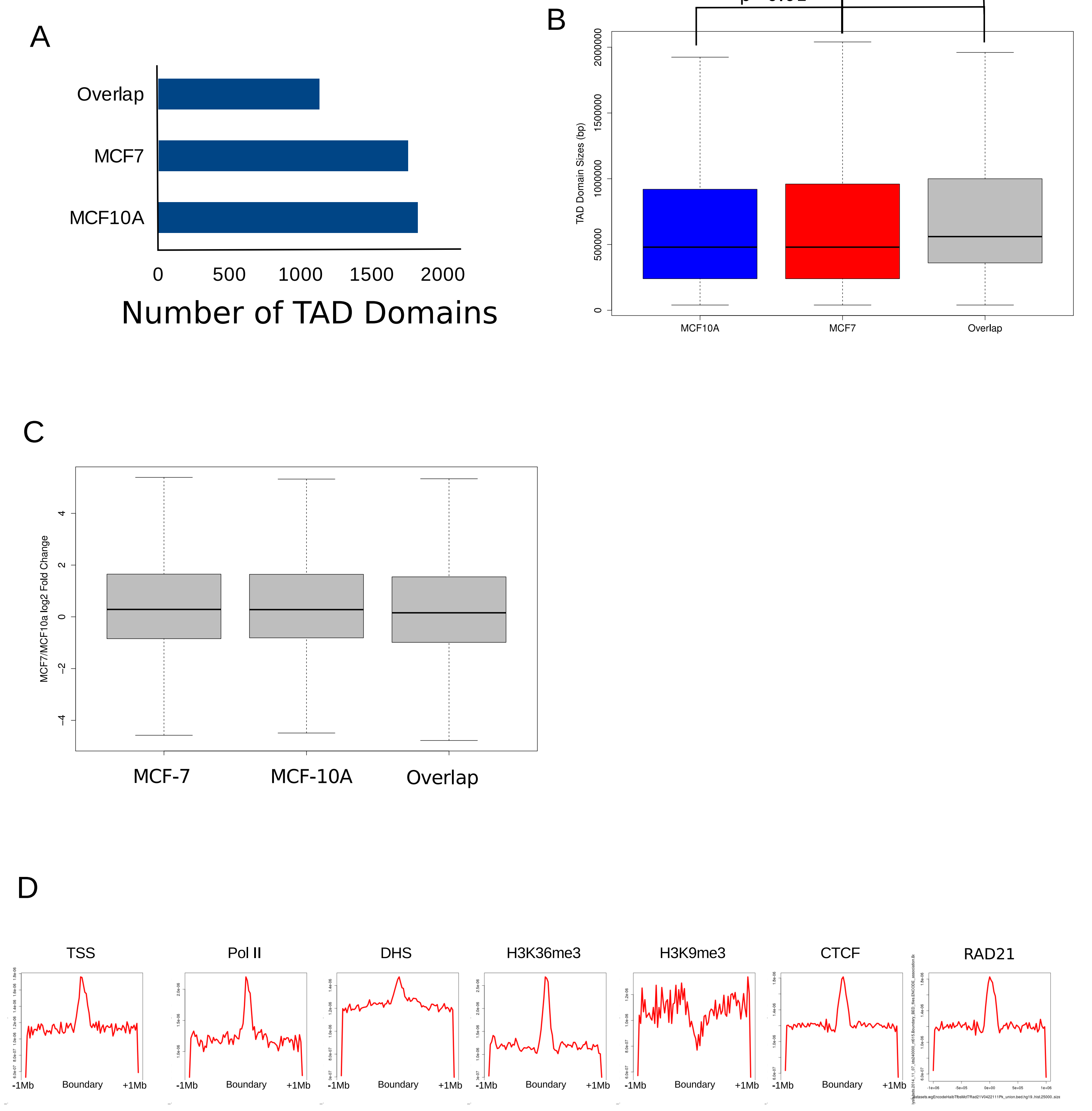

FigureS10– Cell-specific TAD domains do not show enrichment of differentially expressed genes

A) Number of TAD Domains for overlapping, MCF-7 specific and MCF-10A specific TADs

B) Boxplot showing the sizes of TAD domains for MCF-10A specific, MCF-7 specific and overlapping TADs

C) Boxplot showing the MCF7/MCF10A log2 gene expression profiles for MCF-7, MCF-10A and Overlapping TADs

D) Known epigenetic factors that are associated with MCF-7 TAD boundaries

Figure S11

A

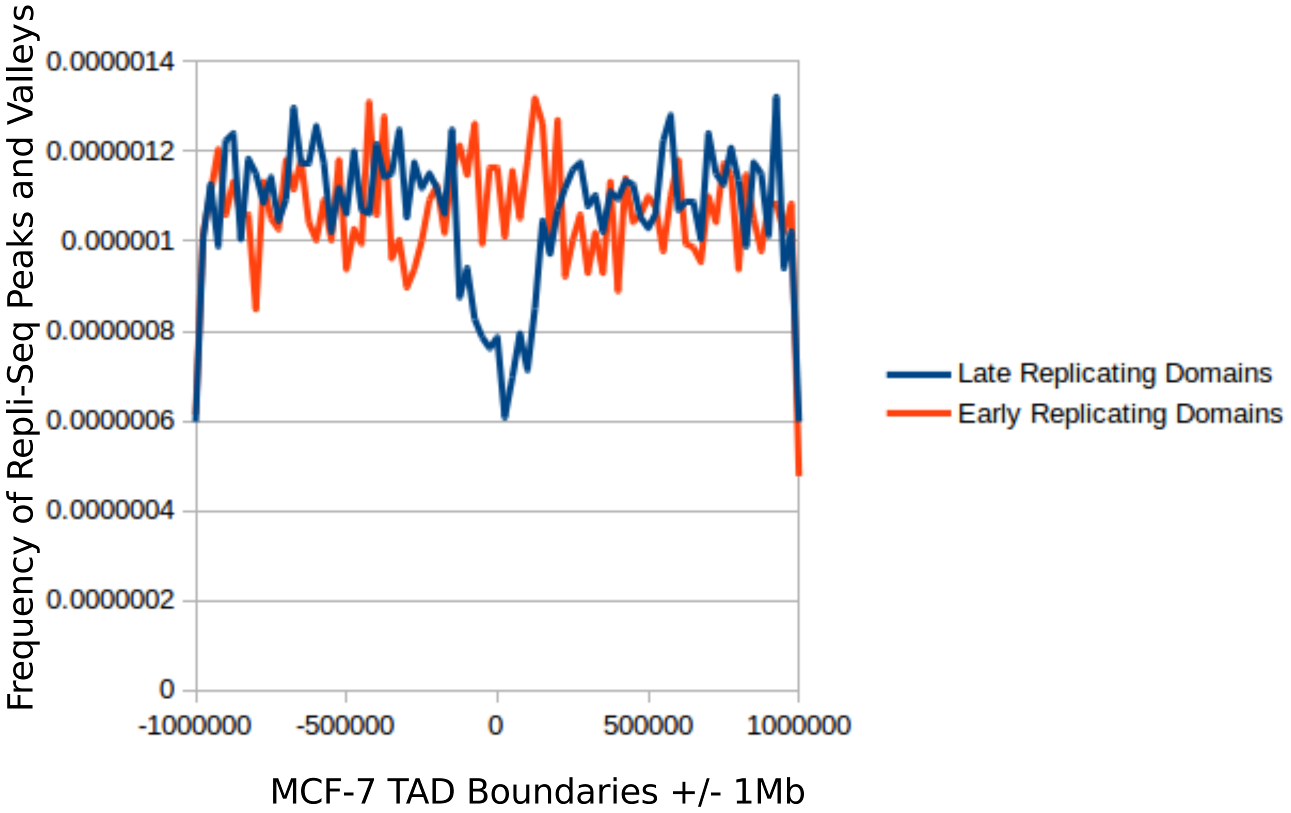

B

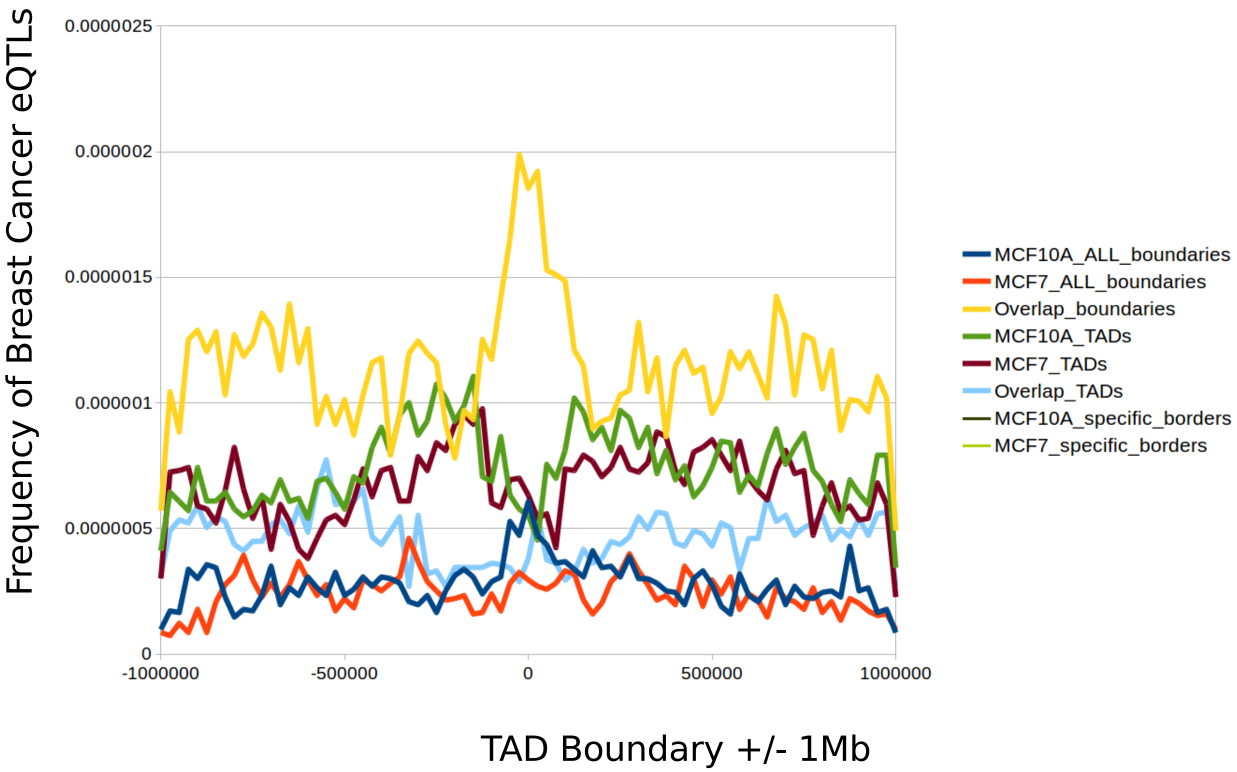

FigureS11 – TAD boundary versus replication timing and eQTL analyses

A) MCF-7 Repli-seq data (Pope et al. 2014) correlation with MCF-7 TAD boundaries

B) Enrichment of Breast Cancer eQTLs (Li et al. 2013) with MCF-7 TAD boundaries as well as domains (TADs)

Figure S12

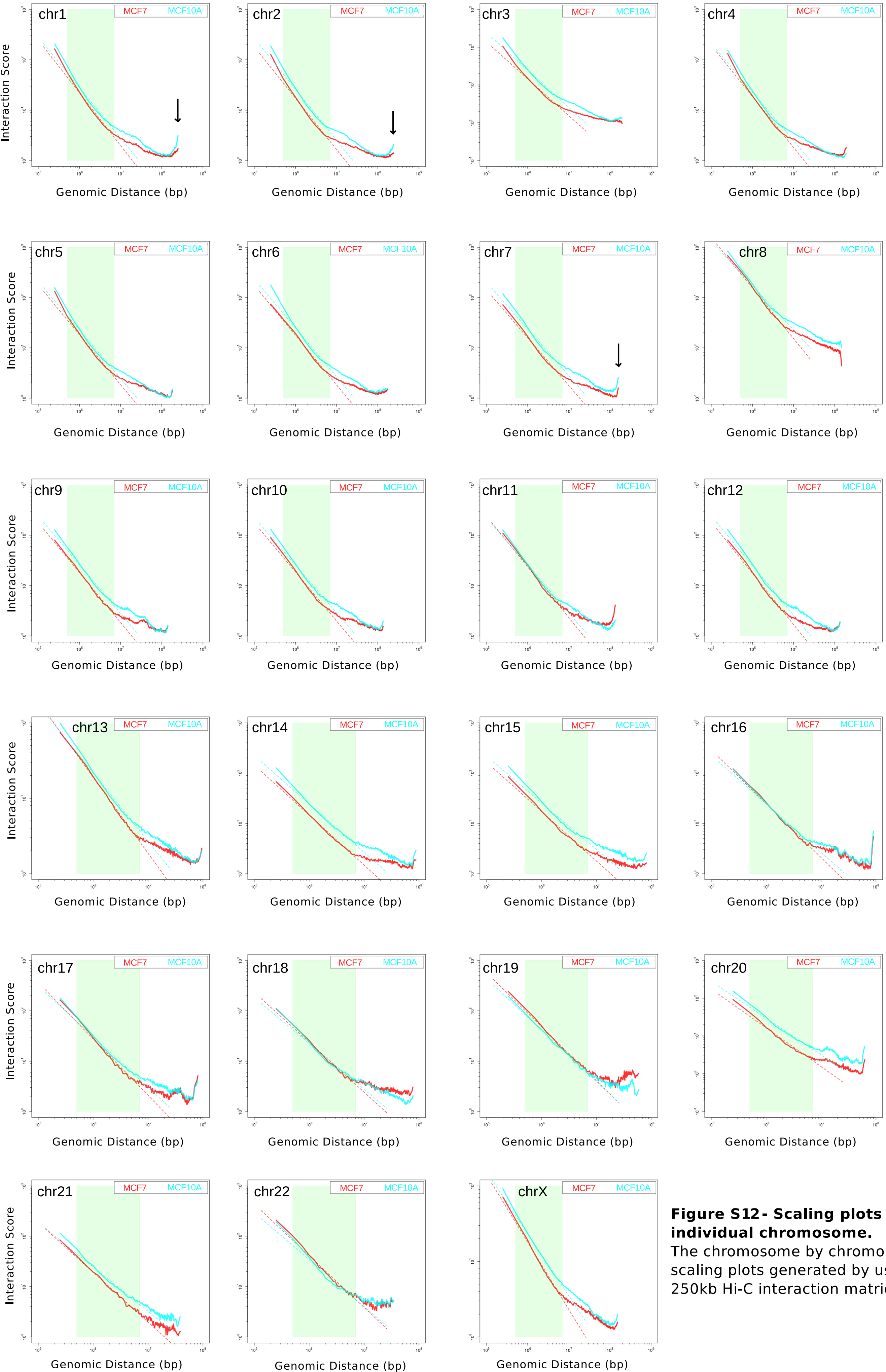

Figure S12 - Scaling plots for each individual chromosome.
The chromosome by chromosome scaling plots generated by using 250kb Hi-C interaction matrices

Figure S13

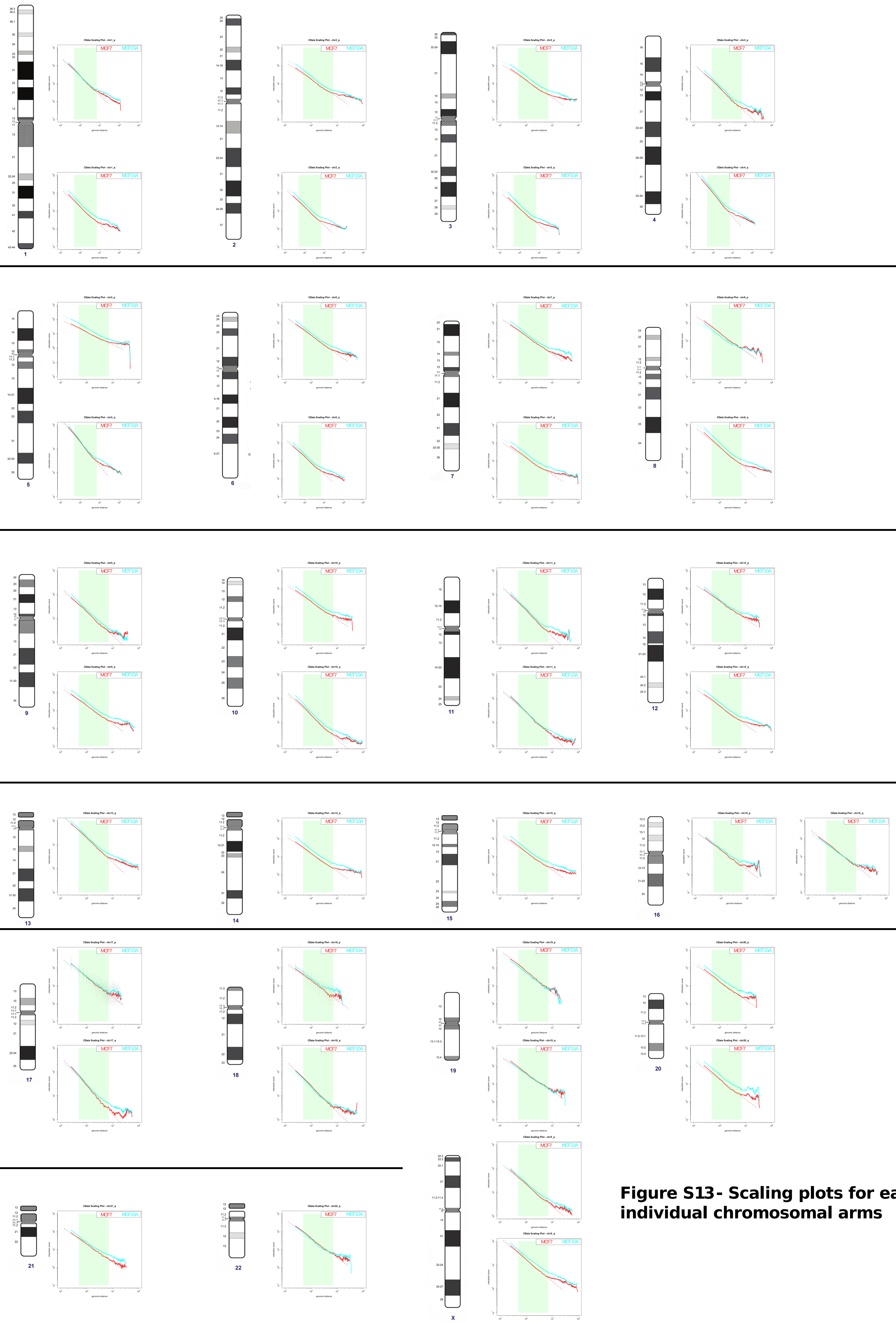

Figure S13- Scaling plots for each individual chromosomal arms

Figure S14

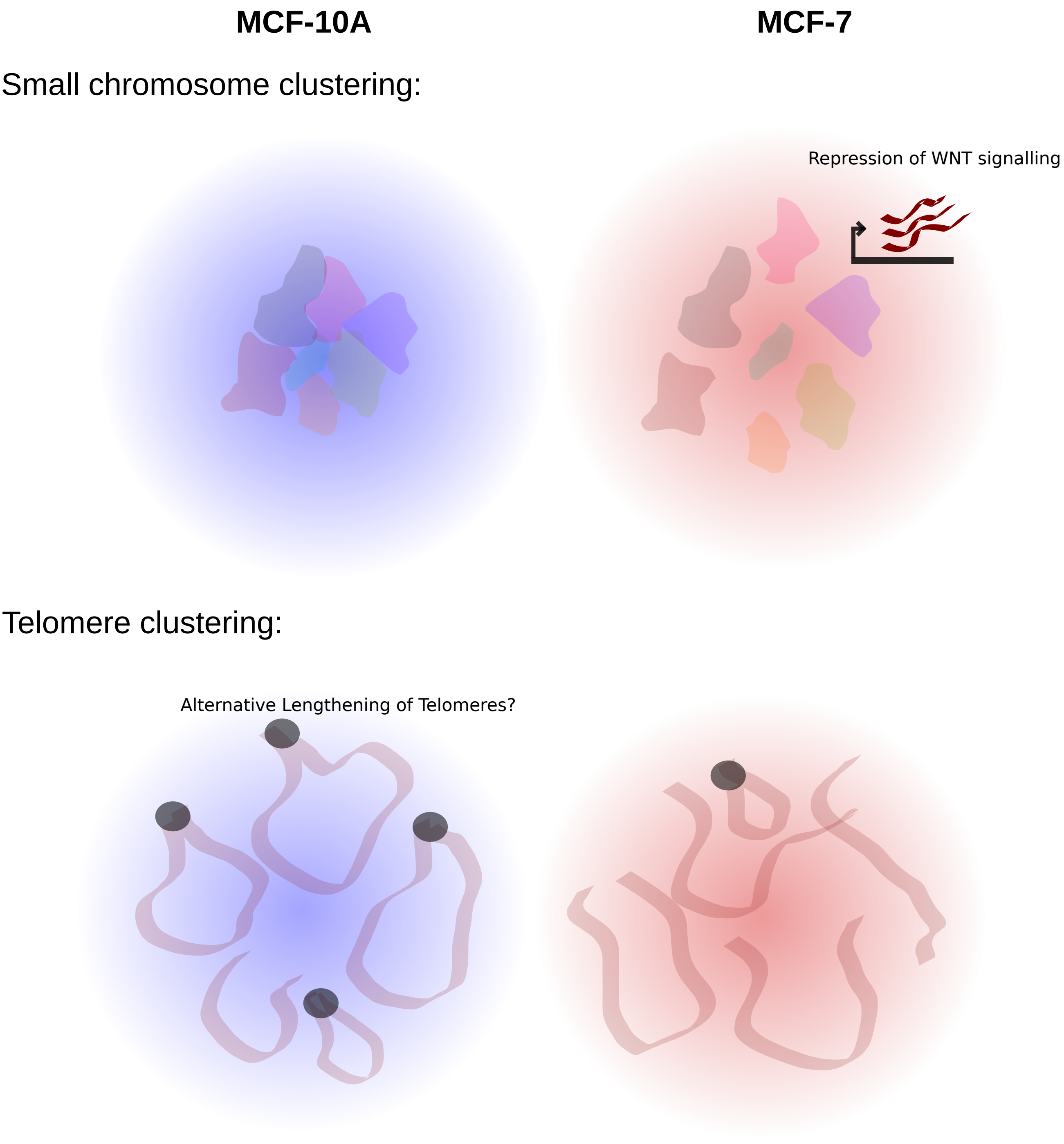

Figure S14 – Summary of the large-scale chromosomal changes between MCF-10A and MCF-7
There are two large-scale changes between the genomes of MCF-10A and MCF-7. First, the inter-chromosomal interactions between the small, gene rich chromosomes are drastically weaker in the MCF-7 breast cancer genome. In concordance with this, there is higher frequency of open genomic compartments, and higher gene expression on chromosomes 16 through 22, especially of the genes related with pathways reflecting the phenotype of the MCF-7 cells. Secondly, the intra-chromosomal associations of the telomeric ends of chromosomes are lost in the MCF-7 genome. This phenomenon may reflect differential telomeric maintenance mechanisms (see Discussion).
